# When you have a hammer, everything looks like a nail: but what kind of hammer is ChatGPT?

**DOI:** 10.1007/s43678-023-00631-x

**Published:** 2024-01-09

**Authors:** Jessalyn K. Holodinsky, Jesse O. Wrenn, Sachin Trivedi, Erik Hess, Eddy Lang

**Affiliations:** 1 Department of Emergency Medicine, Cumming School of Medicine, University of Calgary, Calgary, AB Canada; 2 Department of Community Health Sciences, Cumming School of Medicine, University of Calgary, Calgary, AB Canada; 3 Center for Health Informatics, Cumming School of Medicine, University of Calgary, CWPH 5E36, 3280 Hospital Drive NW, Calgary, AB T2N 4Z6 Canada; 4 Hotchkiss Brain Institute, Cumming School of Medicine, University of Calgary, Calgary, AB Canada; 5 O’Brien Institute for Public Health, Cumming School of Medicine, University of Calgary, Calgary, AB Canada; 6 Alberta Children’s Hospital Research Institute, Cumming School of Medicine, University of Calgary, Calgary, AB Canada; 7 Department of Emergency Medicine, Vanderbilt University Medical Center, Nashville, TN USA; 8 Division of Emergency Medicine, Tennessee Valley Healthcare System VA, Nashville, TN USA; 9 Department of Emergency Medicine, University of Saskatchewan, Saskatoon, SK Canada

ChatGPT (OpenAI Inc., San Francisco, CA), a large language model (LLM), is an impressive piece of technology. The work of Franc-Law et al. examining ChatGPT use in triage is right to consider what this technology can do for emergency physicians, especially as the waiting room gets more and more full, the documentation burden increases, and the crisis of burnout and understaffing continues. The authors present a well-designed study that convincingly tells us that no, ChatGPT cannot perform triage. We commend the authors on their work and on their novel use of the Gauge R and R methodology. However, in the immortal words of Dr. Ian Malcolm when he experienced Jurassic Park for the first time, “Your scientists were so preoccupied with whether they could, they didn't stop to think if they should.” Herein, we will discuss the ever-expanding field of artificial intelligence (AI), reasons for concern in the use of large language models (LLM) in medical decision-making, and areas where LLM may provide value for emergency physicians.

Broadly speaking, AI encompasses computer systems capable of performing tasks that historically required human intelligence such as identifying patterns or making decisions. There are several different AI techniques that have been found to be useful in medical applications including machine learning (ML), deep learning, and natural language processing (NLP). ML algorithms aim to imitate the way humans learn, gradually and with improving accuracy. These algorithms are trained on existing data to make classifications or predictions and perform well with tasks involving pattern and relationship recognition. One example is that of Taylor et al. who utilized several different ML techniques to accurately predict positive urine culture results in individuals visiting the Emergency Department (ED) with symptoms of urinary tract infection [1]. Deep learning can explore more complex patterns in data and has found a use in emergency medicine in imaging analysis. Hwang and colleagues showed that a deep learning algorithm can identify clinically relevant abnormalities on chest X-rays with high sensitivity and specificity [2].

NLP aims to extract structured data from text such as clinical notes or discharge summaries which are typically incomprehensible for machines. This can then be used for classification or prediction, often as a part of another AI method such as a neural network (deep learning). An example pertinent to the ED is when Joseph et al. used a neural network including structured (demographic, vital signs) and unstructured (free text chief complaint notes) triage data to accurately identify patients likely to die or require ICU admission within 24 h [3]. Of note, classical NLP does not produce text as an output but rather uses text as an input.

LLM (like GPT-3, the backbone of ChatGPT) are deep learning algorithms trained on an enormous amount of text data such that they can learn patterns and relationships in language, resulting in the ability to produce language as an output. It’s easy to be inspired while interacting with ChatGPT; it converses fluently and charismatically unlike anything most of us have ever used before. A natural progression would be to try to use it as you would any intelligent agent with seemingly endless memory, knowledge, and speed. We argue, however, that a deeper understanding of its strengths, and more importantly its weaknesses, is of paramount importance prior to implementation in a clinical setting.

GPT-3 was trained on 45 TB of text data including unmoderated content on public websites such as Wikipedia and Reddit [4]. We often advise patients not to go to the internet to make sense of their symptoms—but this is exactly what ChatGPT does. One could perhaps consider a non-LLM-based deep learning classifier trained on hundreds of thousands of human-triaged patient presentations to be an adequate starting point for this type of triage assessment. However, this still removes the important human element of looking at the patient and the ability to interpret a patient’s complaint which is so important in triage.

The ethics of using ChatGPT in healthcare are complicated [5]. ChatGPT may inherit the biases of its training data; studies have enumerated multiple instances of ChatGPT demonstrating gender, racial, political, and religious biases. Furthermore, ChatGPT has a problem with lies or the more common euphemism “hallucinations.” It confidently reports made-up scientific facts, and when asked where the knowledge came from, confidently makes up references. Even when it does faithfully reproduce facts from its training data, these may be incorrect or outdated given the wide range of unverified sources used to train the model. Finally, LLMs are often referred to as “black boxes” because their decision-making process is not transparent. This makes interpreting their responses difficult and also makes it more difficult for researchers to retrain and correct them.

We expect that LLM and other AI technologies will evolve rapidly and that with this, areas for future research or use of LLM in the ED will expand. Perhaps, for now, the best use for LLM does not lie in using it to replace the parts of emergency medicine that require the human element but rather leveraging it to support patients and health care providers operating within the ED. For example, when a patient is discharged from the ED, they may be given verbal or written discharge instructions. Verbal discharge instructions have been well established as insufficient, and the variability of health literacy in our patient population may require individual curation of written documentation. As such, LLM may have the ability to assist in this capacity and optimize discharges. Furthermore, LLM may have used to reduce the burden of administrative tasks. Documentation is an obvious target, so the question naturally becomes “could LLM function as a scribe during patient interviews?” Beyond this, one must ask if LLM could be used to help in communicating with other physicians. Could it scan individual charts to create consultation requests, or even reports to be sent back to primary care providers? Ultimately, the possibilities and potential of where LLM could take us are vast and, if realized and appropriately used, could result in benefits for all end-users of the ED.
